# Efficacy of nitric oxide donors and EDTA against *Pseudomonas aeruginosa* biofilms: Implications for antimicrobial therapy in chronic wounds

**DOI:** 10.1016/j.bioflm.2025.100280

**Published:** 2025-04-15

**Authors:** Aaron Crowther, Gareth LuTheryn, Ramón Garcia-Maset, Maryam Parhizkar, J. Mark Sutton, Charlotte Hind, Dario Carugo

**Affiliations:** a Department of Pharmaceutics, School of Pharmacy, University College London, London, UK; b Institute of Biomedical Engineering, University of Oxford, Oxford, UK; c Nuffield Department of Orthopaedics, Rheumatology and Musculoskeletal Sciences, University of Oxford, Oxford, UK; d Centre for Urological Biology, Division of Medicine, University College London, London, UK; e UK Health Security Agency, Vaccine Development and Evaluation Centre, Porton Down, Salisbury, UK

**Keywords:** Biofilm, Chronic wound, *Pseudomonas aeruginosa*, Nitric oxide, NONOate, EDTA, Ciprofloxacin

## Abstract

Opportunistic pathogen *Pseudomonas aeruginosa* plays a crucial role in chronic wound biofilms, increasing infection's morbidity and mortality. In recent years, the signalling molecule nitric oxide (NO) and chelating agent tetrasodium EDTA (T-EDTA) have been applied therapeutically owing to their multifactorial effects including bacterial killing, biofilm dispersal, and wound healing. However, previous studies assessing NO's antibiofilm efficacy have not considered the variable pH and temperature of the wound environment. Here, pH-dependent NO donors *N*-diazeniumdiolates (NONOates), PAPA NONOate (PA-NO) and Spermine NONOate (SP–NO), and T-EDTA were applied in wound-relevant pH environments (pH 5.5–8.5) and temperatures (32 °C and 37 °C) to *P. aeruginosa* PAO1 biofilms grown for either 24 or 48 h. At 32 °C and pH 7.5, 250 μM PA-NO reduced 24-h biofilm biomass by 35 %. At 37 °C, 250 μM PA-NO and 4 % w/v T-EDTA caused 21 % and 57 % biomass reduction in 24-h biofilms, respectively. In 48-h biofilms, NONOates did not induce significant biomass reduction, while T-EDTA maintained its efficacy with a 64 % reduction. A subsequent experiment investigated the impact of NONOates and T-EDTA as pre-treatments before exposure to ciprofloxacin. Unexpectedly, NONOate pre-treatment decreased ciprofloxacin's effectiveness, resulting in approximately 1-log increase in viable planktonic and biofilm-residing cells compared to ciprofloxacin alone. It was hypothesized that this protective effect might stem from NO-induced decreased cellular respiration, which inhibits reactive oxygen species (ROS)-mediated bactericidal mechanisms. These findings highlight both the potential and complexities of developing effective antimicrobial strategies for chronic wound infections, emphasizing the need for further research to optimize treatment approaches.

## Highlights

• T-EDTA reduced *P. aeruginosa* biofilm in both 24- and 48-h biofilms.
• NONOates, at wound-relevant pH and temperature, were effective in 24-h biofilms.
• Pre-treatment with NONOates reduced ciprofloxacin efficacy.
• Biofilm age significantly impacted antibiofilm treatment outcomes.

## 1 Introduction

Chronic wounds, such as diabetic foot ulcers and pressure sores, present a significant healthcare burden due to their slow healing and high susceptibility to infection [1]. For example, in 2017/18 the UK's National Health Service (NHS) managed more than 3.8 million wounds, spending over £5 billion on unhealed wounds in one year only [2], whilst 10.5 million people were diagnosed with chronic wounds in USA in 2022 (up by 2.3 million from 2014) [3]. A major contributing factor to poor healing is the presence of biofilms, which are complex microbial communities encased in a protective extracellular matrix composed of extracellular polymeric substances (EPS); the most common components being polysaccharides, extracellular DNA (eDNA), and proteins [4]. Biofilms in wounds disrupt the normal wound healing process by causing chronic inflammation and preventing progression to the subsequent stages of healing [5]. Moreover, biofilms result in increased tolerance to antimicrobial agents, which occurs primarily by two mechanisms: 1) presenting a diffusion barrier that shields pathogens from both the host immune response and antimicrobial treatments, and 2) decreasing cellular metabolism thus downregulating many important antibiotic targets [6,7].

One potential therapeutic approach that has gained attention in recent years involves the use of the endogenous signalling molecule nitric oxide (NO). NO plays a vital role in various physiological processes, including wound healing, immune response modulation, and biofilm dispersal [[8], [9], [10]]. The exact mechanism underpinning NO-mediated dispersal remains under-reported; however, Barraud et al. attributed this to enhanced phosphodiesterase (PDE) activity, which decreases cyclic di-GMP (c-di-GMP) levels in *Pseudomonas aeruginosa* [11]. As c-di-GMP levels are paramount in controlling the transition between bacterial motility and sessility, decreasing its concentration can promote a return of biofilm-residing bacteria to a planktonic state, thus enhancing antibiotic efficacy.

Antimicrobial and antibiofilm effects have been reported following exposure to exogenous gaseous NO [[12], [13], [14], [15], [16]]. However, its clinical applicability is limited owing to a poor stability profile, with a half-life of <6 s in aqueous solutions that is likely to reduce further in conditions that are relevant to *in-vivo* applications [17]. Owing to this poor stability profile, the interest in NO-releasing compounds like *N*-diazeniumdiolates (NONOates), which produce NO following pH-dependent decomposition, has grown in recent years. This is mainly due to the possibility of controlling NO release from these compounds, thus providing a potential modality to mitigate issues associated with limited NO stability. However, the efficacy of NONOates against biofilms, particularly mature biofilms found in chronic wounds, remains underreported in the literature. Cai and Webb reported a ∼60–70 % biomass reduction in *P. aeruginosa* PAO1 biofilms (grown statically in M9 media for either 24- or 72-h) following a 2-h treatment with 250 μM Spermine NONOate (SP–NO) [18]. Zhu et al. instead reported a 90 % biomass reduction in *P. aeruginosa* PAO1 biofilms (grown upon shaking in M9 media for 24 h) after 15 min of exposure to 200 μM SP-NO. Although these results reveal the potential of NONOates in antibiofilm treatment, many of the previous studies cannot be directly compared owing to a lack of harmonisation in the methodologies used. Additionally, the use of nutrient-deficient growth media and limited consideration of wound-relevant pH and temperatures, and of their effects on NO release from NONOates, highlight an area that merits further research. An alternative antibiofilm agent, ethylenediaminetetraacetic acid (EDTA), is currently used in wound dressings and venous lock catheters, owing to its potent antimicrobial and antibiofilm effects [[19], [20], [21], [22]]. EDTA acts against the biofilm matrix and the bacterial cell wall by chelating essential cations, thus impairing cellular function and degrading EPS components [23].

Here, the antibiofilm effects of tetrasodium EDTA (T-EDTA) and two candidate NONOates (SP–NO and PAPA NONOate (PA-NO)) were assessed. Additionally, the potentiation of antibiotic efficacy following a “pre-treatment” phase with antibiofilm agents was assessed in wound-relevant pH and temperature environments for the first time. The experimental conditions evaluated included different levels of pH (which is variable throughout the wound healing cycle) and temperature (which appears lower than physiological in wounds). Treatment efficacy was also evaluated in biofilms cultured for both 24- and 48-h, to assess applicability in mature biofilms. Findings from this study can contribute towards the development of novel antibiofilm agents, suitable for adjuvant therapy with clinically relevant antibiotics against wounds of varying chronicity.

## 2 Materials and methods

### 2.1 Selection of NONOate candidates

Spermine NONOate (SP–NO) and propylamine propylamine (PAPA) NONOate (PA-NO) (Cambridge Biosciences, UK) were selected for initial studies due to their favourable half-life characteristics (15 and 39 min, respectively, at 37 °C and pH 7.4) [24,25] and previously reported efficacy as antibiofilm agents [18,26,27]. Vials containing NONOate powder were flushed with nitrogen after use and stored at −80 °C. Once opened, vials were discarded after 1 month to account for their limited stability after exposure to air and moisture.

### 2.2 NONOate characterisation

#### 2.2.1 Griess assay

A nitric oxide colorimetric assay kit (Abcam, UK) containing assay buffer, nitrate standard, nitrate reductase, enzyme cofactor, enhancer, and Griess reagents was used to confirm pH-dependent release of NO from candidate NONOates. Reagents were prepared and stored according to the manufacturer's instructions. NONOate stock solutions (2.5 mM) were prepared in 0.01 M sodium hydroxide (NaOH) and kept on ice (∼4 °C) during experiments, ensuring that the final concentrations fell within the assay range (1–10 nmol/well). Buffer solutions (MES buffer (pH 5.5), PBS (pH 7.5), or TAPS buffer (pH 8.5) (Sigma-Aldrich, USA)) were adjusted to the desired pH at 35 °C using 1 M NaOH or HCl (Sigma-Aldrich, USA). Fresh 1 mM nitrate standards were prepared on the day of the experiment and used within 4 h of preparation. For negative controls, 85 μL of buffer solution was added to wells of a flat-bottom, transparent 96-well microplate (Corning, Netherlands). Test samples consisted of NONOate in pH-adjusted buffer, incubated for 30 min before 85 μL aliquots were added to the microplate.

For all wells (standards, controls, and tests), 5 μL of nitrate reductase and enzyme cofactor were added, followed by a 60-min incubation at room temperature. Next, 5 μL of enhancer was added, followed by 10 min of incubation, then 50 μL each of Griess reagent R1 and R2 were added. Absorbance was measured at 540 nm using a microplate reader (SPARK, Tecan). Mean absorbance values were calculated from duplicates, and the blank-corrected mean values were plotted to generate a standard curve. Nitrite concentration was determined using the slope of the linear calibration curve. All experiments were conducted in triplicate, yielding six absorbance values per condition tested.

#### 2.2.2 Determination of NONOate degradation kinetics via UV–visible spectroscopy

To determine the degradation profile of candidate NONOates, UV–visible spectroscopy was used to detect the presence of intact NONOate. For degradation kinetics analysis, 250 μM PA-NO or SP-NO was prepared in MES buffer (pH 5.5), PBS (pH 7.5), or TAPS buffer (pH 8.5) (Sigma-Aldrich, USA). Immediately after preparation, 200 μL of each solution was transferred into three wells of a UV-transparent 96-well microplate (Thermo Fisher Scientific, USA). Absorbance was measured at 250 nm for PA-NO and 252 nm for SP-NO at 5-min intervals, for 4 h at 32 °C [24,25].

### 2.3 Bacterial species and culture conditions

*P. aeruginosa* (PAO1) was grown on Lysogeny broth (LB) agar plates by streaking a glycerol stock of PAO1 and incubating at 37 °C for 18–24 h. Plates were placed in the refrigerator (4–6 °C) and were considered viable for up to 3 weeks. To grow an overnight broth culture, a sterile inoculating loop was used to select three identical colonies from the plate and inoculate 5 mL of LB broth; this was statically incubated overnight at 37 °C.

#### 2.3.1 Biofilm growth conditions

*P. aeruginosa* PAO1 biofilms were grown in sterile, transparent, flat-bottom, 96-well microtiter plates (Corning, Netherlands) by inoculating each well with 150 μL of culture diluted to an initial inoculum of ∼1 x 10^5^ CFU/mL. Each single replicate consisted of six wells and each condition was carried out in triplicate; therefore, eighteen wells were prepared for each condition. All peripheral wells were filled with 200 μL sterile water to prevent differential growth across the plate due to evaporation. Plates were incubated for 24 or 48 h at 37 °C to allow successful biofilm growth.

### 2.4 Evaluating antibiofilm efficacy of treatments

#### 2.4.1 Crystal violet assay

Crystal violet (CV) was used as a method to quantify total biomass as it stains both live and dead cells, as well as negatively charged surface molecules and polysaccharides in the biofilm matrix [28,29]. After biofilm growth, planktonic bacteria were gently removed using a multichannel micropipette, without disrupting the biofilm. Wells were washed once with sterile water to remove any remaining planktonic bacteria before adding 150 μL of control or treatment solution. Untreated control biofilms were exposed to LB broth only and treatment wells were treated with NONOate in 1:1 LB broth and the relevant buffer solution for the desired pH (MES, PBS, or TAPS) or 4 % w/v T-EDTA (Merck, Germany). NONOate solutions were assessed at a range of concentrations and pH levels (5.5, 7.5, and 8.5). Plates were then incubated for 2 h at 37 °C. Control and treatment solutions were then gently removed from the wells and washed once with sterile water.

150 μL of 0.1 % (v/v) CV (Sigma-Aldrich, USA) was then added to all wells containing biofilm and left for 15 min. CV was then removed from the wells, which were washed 2–3 times with sterile water to ensure no excess stain remained in the wells. Plates were then inverted on paper towel to remove excess liquid from the wells and left on the bench overnight to air-dry.

To quantify biofilm biomass, bound CV was solubilised by adding 150 μL of 30 % (v/v) acetic acid to all stained wells and left at room temperature for 30 min. 100 μL from all stained wells was then transferred to a new 96-well microplate, including 6 blank wells containing 30 % (v/v) acetic acid only. The sample absorbance was then measured at 584 nm on the microplate reader. A mean absorbance value of six wells was taken as one technical replicate; a total of three technical replicates and three biological replicates were carried out.

#### 2.4.2 Fluorescence microscopy

*P. aeruginosa* PAO1 biofilms were grown in 0.8 mm ibidi μ-Slide channel slides (ibidi, Germany) that had been pre-coated with 50 μg/mL of fibronectin (Corning, Netherlands) to enhance bacterial adhesion [30]. The fibronectin solution was incubated in the channels for 60 min at room temperature and allowed to air dry before 300 μL of *P. aeruginosa* PAO1 cells were seeded (∼1 x 10^5^ CFU/mL). The biofilms were incubated for 24 h at 37 °C. After the biofilm formation period, the channels were gently washed with sterile PBS to remove non-adherent bacteria. Biofilms were then treated with 300 μL of LB (untreated control), 4 % w/v T-EDTA, 250 μM PA-NO, or 250 μM SP-NO for 2 h at 37 °C. Treatment was then removed and biofilms were gently washed with sterile PBS.

Following washing, the biofilms were stained using the LIVE/DEAD® FilmTracer™ Bacterial Viability Kit (Thermo Fisher Scientific, USA). The staining solution, consisting of SYTO 9 and propidium iodide (PI) dyes, was prepared according to the manufacturer's instructions, and 300 μL was added to each channel. The biofilms were incubated with the stain for 30 min at room temperature, protected from light. After staining, the biofilms were imaged using a Nikon Ti2 inverted microscope with a 10× Plan Fluor objective. 3 × 3 tile scans were taken from three distinct locations within each biofilm, covering multiple regions for comprehensive imaging. The images were then stitched together, and one representative image per experimental condition was selected for reporting.

### 2.5 Assessing the viability of bacteria in 24- and 48-h biofilms

Biofilms were grown as described previously; briefly, overnight cultures were prepared by inoculating 5 mL of LB broth. 150 μL of *P. aeruginosa* PAO1 was seeded in each well (initial inoculum = ∼1 x 10^5^ CFU/mL). Plates were then incubated at 37 °C for 24 or 48 h. In 48-h biofilms with a half-media change, 75 μL of the planktonic culture was replaced with 75 μL of fresh sterile LB broth after 24 h. After incubation, biofilms were dispersed in fresh sterile LB broth by vigorous pipetting. Resazurin was then used to assess metabolic activity by applying 15 μL of alamarBlue™ Cell Viability Reagent (Thermo Fisher Scientific, USA) and plates were allowed to incubate in the dark for 1 h at 37 °C. After incubation, fluorescence intensity was read by exciting at 550 nm and capturing emissions at 600 nm using a plate reader. Colony-forming units (CFUs) were counted by pooling 10 μL from all six replicates and serially diluting this 10-fold from 10^−1^ to 10^−7^. Three 10 μL aliquots of each dilution were then spot plated on LB agar plates and allowed to incubate for 24 h. After incubation, CFUs were counted, and the corresponding CFU/mL was calculated.

### 2.6 Antimicrobial susceptibility testing

The minimum inhibitory concentration was determined for ciprofloxacin, gentamicin, vancomycin, and T-EDTA against *P. aeruginosa* PAO1. The broth microdilution test recommended by the European Committee on Antimicrobial Susceptibility Testing (EUCAST) was carried out as follows [31]. Antibiotics were prepared by first producing a 10 mg/mL stock in water or DMSO, depending on solubility, and subsequently diluting this to a working concentration in sterile cation-adjusted Mueller-Hinton broth (caMHB). Antibiotics were then serially diluted two-fold in 96-well plates vertically to assess 8 concentrations in total; 100 μL of antibiotic solution was mixed with 100 μL caMHB. Bacterial cultures were grown overnight in 5 mL caMHB at 37 °C, in shaking conditions (180 rpm). Cultures were then diluted to give a suspension with an OD600 of 0.1 AU, which was equivalent to ∼1 x 10^8^ CFU/mL; this was then diluted 1:100 to give ∼1 x 10^6^ CFU/mL for inoculation. 100 μL of this bacterial suspension was added to each well containing antibiotic to give a final bacterial concentration of ∼5 x 10^5^ CFU/mL. Four positive control wells contained 100 μL of bacterial suspension plus 100 μL caMHB, whilst four negative control wells contained 200 μL of caMHB only. 96-well plates were then incubated at 37 °C for 24 h, after which metabolic activity was assessed by resazurin staining (as described in Section 2.3.6). Each condition was tested with three biological replicates using three separate overnight cultures, whilst each 96-well plate contained three technical replicates.

### 2.7 Assessment of antibiotic potentiation by CFU counting

*P. aeruginosa* PAO1 biofilms were grown for 24 h as previously described. Planktonic cultures were then removed from the wells and biofilms were washed once with 150 μL sterile PBS. After washing, 150 μL of antibiofilm treatments (LB for control, 4 % w/v T-EDTA, 250 μM PA-NO, or 250 μM SP-NO) were applied and incubated at 37 °C for 2 h, followed by another wash with 150 μL sterile PBS. Following this washing step, 150 μL of 0.25 μg/mL ciprofloxacin (corresponding to MIC) was applied to biofilms and allowed to incubate for 24 h at 37 °C. Following incubation, the treatment supernatant was removed (all six wells were pooled for each technical replicate), placed into 1.5 mL reaction tubes, and centrifuged at 4000 rpm for 10 min to produce a pellet of cellular material. The centrifuged supernatant was removed, and the pellet was resuspended in 900 μL of fresh LB broth by vortex mixing for 30 s. Biofilms were washed twice with 150 μL sterile PBS and resuspended in fresh LB broth by placing in a sonication bath for 10 min. All six wells were also pooled for each technical replicate. For enumeration of viable cells, both supernatant and biofilm suspensions were serially diluted 10-fold to 10^−6^. The track dilution method was then carried out by placing 10 μL of each dilution on an LB agar plate, which was allowed to flow across the surface creating tracks of culture [32]. Plates were then incubated overnight after which CFUs were counted.

### 2.8 Statistical analyses

Statistical analyses were conducted using GraphPad Prism 10.3.1. For Griess assay experiments, comparisons were made using two-way analysis of variance (ANOVA) with Šídák's multiple comparisons test to assess the statistical significance of differences between pH levels and between different NONOate types. For crystal violet biomass studies, one-way ANOVA with Tukey's multiple comparisons was carried out. For combination treatment studies assessing viable cells, pairs of Welch's *t*-test was used to account for unequal variances between groups. For all analyses, a threshold value for significance was set at < 0.05; where ∗ = P < 0.05, ∗∗ = P < 0.005, ∗∗∗ = P < 0.0005, and ∗∗∗∗ = P < 0.0001.

## 3 Results

### 3.1 NO detection and quantification

#### 3.1.1 Characterisation of NO release from candidate NONOates using a colorimetric assay

The NO release from candidate NONOates was investigated at different pH levels by using a colorimetric assay (Griess) to gain further insight into how the pH environment can influence release kinetics.

**Fig. 1:**
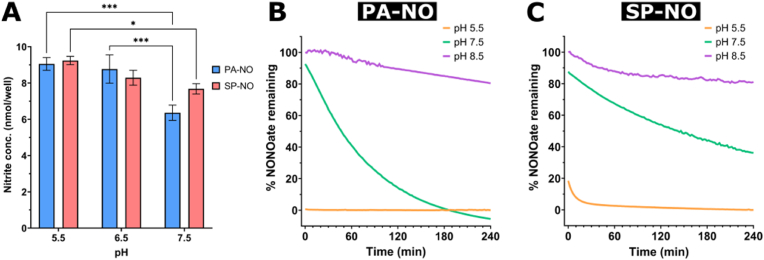
(A) Nitrite concentration (nmol/well) produced by PAPA NONOate (PA-NO; blue bars) and Spermine NONOate (SP–NO; red bars) determined by the colorimetric Griess assay (samples were incubated at 35 °C). Two-way ANOVA was carried out for statistical comparison. Statistical differences are indicated by: ∗ = p < 0.05, ∗∗ = p < 0.01, ∗∗∗ = p < 0.001, and ∗∗∗∗ = p < 0.0001. Error bars indicate standard deviation of the mean. Experiments were carried out in triplicate (n = 3). The kinetic degradation curves for (B) PA-NO and (C) SP-NO at pH 5.5 (orange line), 7.5 (green line), and 8.5 (purple line) (samples were incubated at 32 °C). (For interpretation of the references to color in this figure legend, the reader is referred to the Web version of this article.)

Generally, the results from the Griess assay showed a trend of decreasing nitrite concentration as the pH of the solution was increased in both NONOate groups; this indicates a decrease in NO concentration in higher pH environments (Fig. 1A). SP-NO showed no significant difference in the detected nitrite concentration between the pH 5.5 and pH 6.5 groups or between the pH 6.5 and pH 7.5 groups; however, a significant difference (P < 0.05) was determined between the pH 5.5 and pH 7.5 groups (Fig. 1A). PA-NO also showed no significant difference in nitrite concentration between the pH 5.5 and pH 6.5 groups; however, PA-NO did show a significant difference (P < 0.001) in the detected nitrite concentration between the pH 6.5 and pH 7.5 groups and between the pH 5.5 and pH 7.5 groups (Fig. 1A). Statistical analyses were also carried out to assess any difference in nitrite concentration detected between NONOates at the same pH environment; however, no significant difference was found.

#### 3.1.2 Determining the effect of pH on NONOate degradation kinetics

The presence of intact NONOate molecules over time was assessed by measuring absorbance at their respective *λ*_max_ values. The results showed an increase in the rate of NONOate degradation as the pH of the release media was decreased. PA-NO and SP-NO both showed slow, sustained degradation at pH 8.5, showing 19.5 % and 18.9 % degradation after 4 h, respectively (Fig. 1B and C). The release profiles observed at pH 7.5 clearly indicated first-order NO release kinetics, with PA-NO achieving near-full degradation after 4 h and SP-NO showing 64 % degradation after 4 h. At pH 5.5, decomposition appeared to occur markedly quicker in both NONOates, with PA-NO showing a very short period of sharp decrease initially and reaching 100 % degradation within the first 5 min of the experiment (Fig. 1B). SP-NO also showed rapid degradation at pH 5.5 for the initial 10 min, before plateauing and reaching 99 % degradation after 60 min (Fig. 1C).

### 3.2 The antibiofilm efficacy of NONOates

#### 3.2.1 24-hour biofilms

**Fig. 2:**
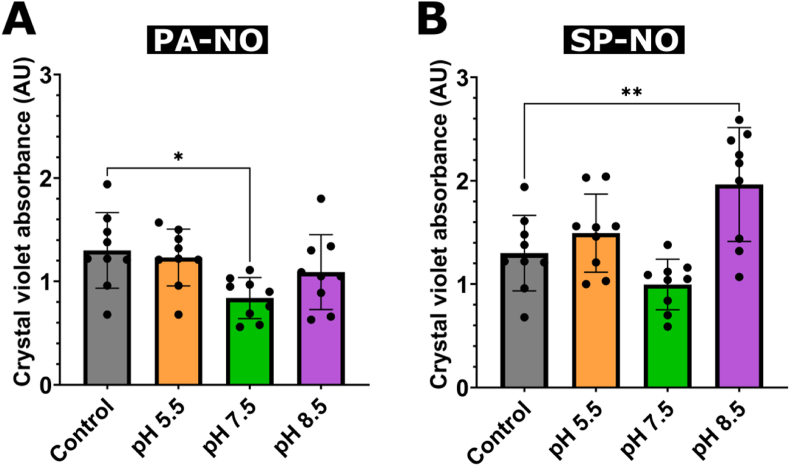
Total biofilm biomass (crystal violet) of *P. aeruginosa* PAO1 biofilms grown for 24 h after 2-h treatment with (A) 250 μM PAPA NONOate (PA-NO), or (B) 250 μM Spermine NONOate (SP–NO) at either pH 5.5 (orange bars), 7.5 (green bars), or 8.5 (purple bars). The untreated control (grey bars) was supplemented with LB broth for 2 h. Samples were incubated at 32 °C. One-way ANOVA was carried out for statistical comparison. Statistical differences are indicated by: ∗ = p < 0.05, ∗∗ = p < 0.01, ∗∗∗ = p < 0.001, and ∗∗∗∗ = p < 0.0001. Error bars indicate standard deviation of the mean. Experiments were carried out with 3 biological replicates and 3 technical replicates (n = 9). (For interpretation of the references to color in this figure legend, the reader is referred to the Web version of this article.)

Initially, NONOates were applied to 24-h biofilms for 1 h at varying concentrations and pH levels at the wound-relevant temperature of 32 °C; however, no significant reductions were determined compared to the control (Fig. S1). Considering the lack of dispersal after 1 h of treatment, antibiofilm efficacy was assessed after treatment with 250 μM PA-NO and 250 μM SP-NO for 2 h at 32 °C in various wound-relevant pH environments (i.e., pH 5.5, 7.5, and 8.5), to confirm suitability for future *in-vivo* applications (Fig. 2). Whilst PA-NO showed a significant biomass reduction of 36 % (P < 0.05) at pH 7.5 compared to control, no significant reduction was observed at pH 5.5 or 8.5 (Fig. 2A). SP-NO showed no significant reduction in biomass at pH 5.5 or 7.5; however, a significant increase (of 51 %) was determined at pH 8.5 (P < 0.0001) (Fig. 2B).

**Fig. 3:**
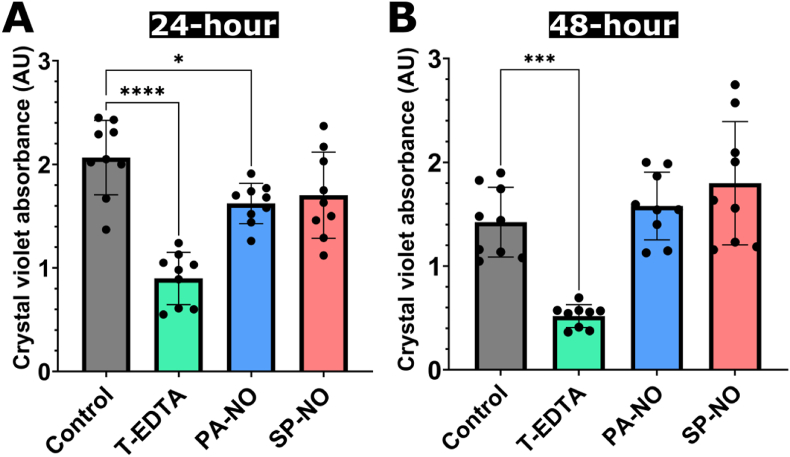
Total biofilm biomass (crystal violet) of *P. aeruginosa* PAO1 biofilms grown for 24 h (A) and 48 h (B) treated for 2 h with either 4 % w/v tetrasodium EDTA (T-EDTA; green bars), 250 μM PAPA NONOate (PA-NO; blue bars), or 250 μM Spermine NONOate (SP–NO; red bars). The untreated control (grey bars) was supplemented with LB broth for 2 h. Samples were incubated at 37 °C. One-way ANOVA was carried out for statistical comparison. Statistical differences are indicated by: ∗ = p < 0.05, ∗∗ = p < 0.01, ∗∗∗ = p < 0.001, and ∗∗∗∗ = p < 0.0001. Error bars indicate standard deviation of the mean. Experiments were carried out with 3 biological replicates and 3 technical replicates (n = 9). (For interpretation of the references to color in this figure legend, the reader is referred to the Web version of this article.)

**Fig. 4:**
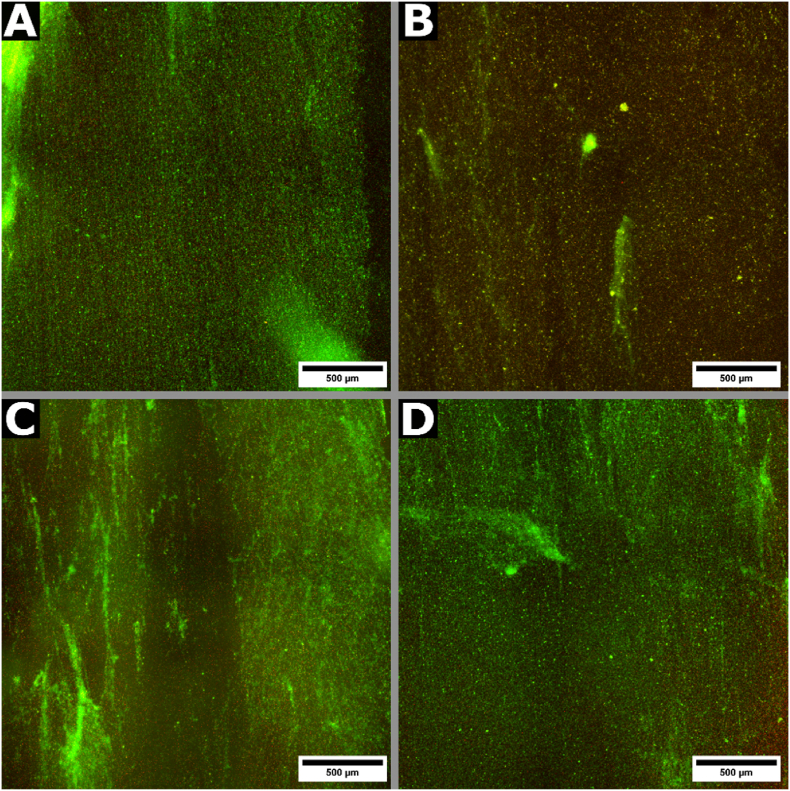
Fluorescence microscopy images of a SYTO9- (green) and PI- (red) stained *P. aeruginosa* PAO1 biofilm (grown for 24 h) after 2 h of treatment with (A) LB broth (untreated control), (B) 4 % w/v tetrasodium EDTA (T-EDTA), (C) 250 μM PA-NO, or (D) 250 μM SP-NO. Samples were incubated at 37 °C. (For interpretation of the references to color in this figure legend, the reader is referred to the Web version of this article.)

Further experiments were carried out at a greater treatment incubation temperature of 37 °C, for comparison with previous published studies in the literature [18,33]. 4 % w/v T-EDTA was also included in these experiments to evaluate an additional antibiofilm agent with a distinct mechanism of action to NO [23]. 4 % w/v T-EDTA caused a significant reduction in biomass (P < 0.0001) compared to control, with a percentage reduction value of 57 % (Fig. 3, Fig. 4B). 250 μM PA-NO showed a significant reduction in biomass compared to control (P < 0.001) (Fig. 4C), with a percentage reduction of 21 %, whilst 250 μM SP-NO showed no significant reduction in biomass (Fig. 3, Fig. 4D).

#### 3.2.2 48-hour biofilms

In order to assess NONOate efficacy against more mature biofilms, the bacterial culture time was increased to 48 h. It was also decided to assess NONOates at pH 7.5 only, as this was observed to be the most effective against 24-h biofilms. Interestingly, neither 250 μM PA-NO nor SP-NO showed a significant difference compared to control (P > 0.05), whilst 4 % w/v T-EDTA showed a significant biomass reduction (P < 0.001) of 64 % (Fig. 3B). The mean absorbance value of the untreated control biofilm was also observed to be 32 % lower in 48-h biofilms compared to 24-h biofilms (2.07 *vs* 1.42 AU, for 24- and 48-h biofilms, respectively), indicating that less biomass was present following a longer growth period.

### 3.3 Comparing the viability of bacterial cells in 24- and 48-h biofilms

**Fig. 5:**
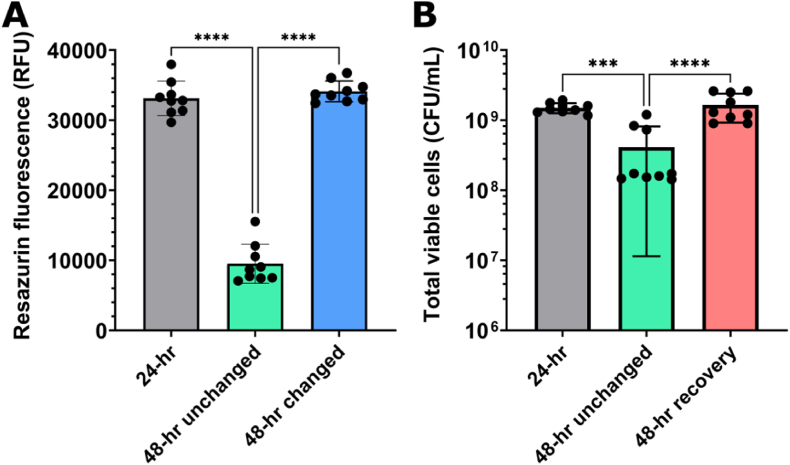
(A) Metabolic activity by resazurin staining of *P. aeruginosa* PAO1 biofilms grown for 24 h (grey bar), 48 h (green bar), or 48 h with a half-media change after 24 h (blue bar). (B) Total viable cells (colony forming units per mL; CFU/mL) from PAO1 biofilms grown for 24 h (grey bar), 48 h (green bar), or 48 h with an addition at 24 h of fresh LB broth (red bar). Samples were incubated at 37 °C. One-way ANOVA was carried out for statistical comparison. Statistical differences are indicated by: ∗ = p < 0.05, ∗∗ = p < 0.01, ∗∗∗ = p < 0.001, and ∗∗∗∗ = p < 0.0001. Error bars indicate standard deviation of the mean. Experiments were carried out with 3 biological replicates and 3 technical replicates (n = 9). (For interpretation of the references to color in this figure legend, the reader is referred to the Web version of this article.)

Due to the lack of antibiofilm efficacy of NONOates on *P. aeruginosa* PAO1 biofilms grown for 48 h at 37 °C, it was hypothesized that the cells residing in the biofilm may have been less metabolically active than those grown for 24 h. To assess this, resazurin staining was used to compare metabolic activity. 24-h biofilms showed a mean fluorescence value of 33120 RFU, which reduced significantly (P < 0.0001) in 48-h biofilms with a mean fluorescence value of 9514 RFU (Fig. 5A). Additionally, to determine if the difference in metabolic activity was caused by nutrient deficiency, half of the growth media in 48-h biofilms was replenished after 24 h. 24 h after the media change, there was no significant difference (P > 0.05) in resazurin fluorescence intensity of 48-h biofilms with a half-media change when compared to 24-h biofilms, with a mean fluorescence value of 34115 RFU (Fig. 5A).

As it was unclear whether the difference in metabolic activity was owed to cell death or cell dormancy, the number of viable cells present in both 24- and 48-h biofilms were compared. A significant difference was determined (P < 0.0001), observing ∼1.5 x 10^9^ CFU/mL and ∼4.1 x 10^8^ CFU/mL in 24- and 48-h biofilms, respectively (Fig. 5B). An additional experimental group was included to attempt to revive the cells after 48 h to determine if they were predominantly dormant or dead, by adding fresh sterile LB broth and allowing to incubate for further 24 h. After incubation, the total viable count was not significantly different (P > 0.05) to that observed in 24-h biofilms, showing ∼1.7 x 10^9^ CFU/mL (Fig. 5B).

### 3.4 Antibiotic potentiation effects of NONOate treatment

#### 3.4.1 Minimum inhibitory concentration (MIC) testing

To aid the antibiotic selection process and provide a baseline when assessing antimicrobial susceptibility in biofilms, broth microdilution was used to determine the MIC of three clinically relevant antibiotics (ciprofloxacin, gentamicin, and vancomycin) and candidate antibiofilm T-EDTA against *P. aeruginosa* PAO1. The MIC of vancomycin was determined to be higher than 32 μg/mL (above resistance breakpoint), whilst ciprofloxacin and gentamicin showed MIC values of 0.25 and 4 μg/mL, respectively (Fig. S2). The MIC of T-EDTA was determined to be 0.5 % w/v. Owing to its potent efficacy and clinical relevance, ciprofloxacin was selected as the candidate antibiotic for further assessment.

#### 3.4.2 Assessment of antibiotic potentiation effects of combination treatments

**Fig. 6:**
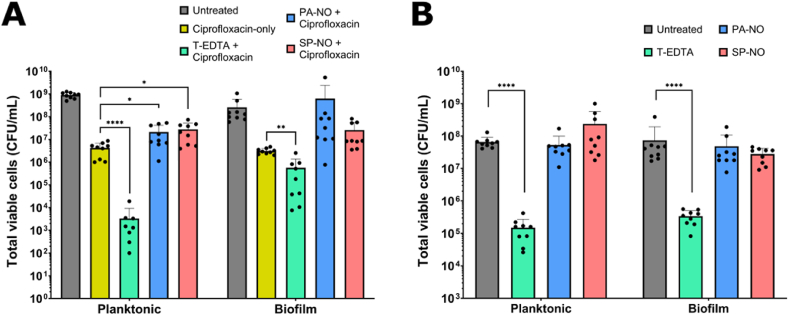
(A) The number of viable cells (colony forming units per mL; CFU/mL) present in 24-h PAO1 biofilms after treatment with 0.25 μg/mL ciprofloxacin for 24 h (yellow bar) or after a 2-h pre-treatment with 4 % w/v tetrasodium EDTA (T-EDTA; green bar), 250 μM PAPA NONOate (PA-NO; blue bar), or 250 μM Spermine NONOate (SP–NO; red bar) followed by 24 h of 0.25 μg/mL ciprofloxacin. (B) The number of viable cells present in 24-h PAO1 biofilms after a 2-h treatment with 4 % w/v T-EDTA (green bar), 250 μM PA-NO (blue bar), or 250 μM SP-NO (red bar). Samples were incubated at 37 °C. Multiple Welch's t-tests were carried out for statistical comparison. Statistical differences are indicated by: ∗ = p < 0.05, ∗∗ = p < 0.01, ∗∗∗ = p < 0.001, and ∗∗∗∗ = p < 0.0001. Error bars indicate standard deviation of the mean. Experiments were carried out with 3 biological replicates and 3 technical replicates (n = 9). (For interpretation of the references to color in this figure legend, the reader is referred to the Web version of this article.)

Ciprofloxacin was applied at MIC (i.e., 0.25 μg/mL) either alone for 24 h or following a 2-h pre-treatment with biofilm dispersal agents (i.e., 250 μM PA-NO, 250 μM SP-NO, or 4 % w/v T-EDTA) for 24 h. Ciprofloxacin showed a ∼2.5-log and ∼2-log reduction in viable cells compared to control in the supernatant and the biofilm, respectively, whilst T-EDTA showed ∼5-log and ∼3-log reductions in supernatant and biofilm, respectively (Fig. 6A). T-EDTA followed by ciprofloxacin treatment was the only combination that enhanced the efficacy of ciprofloxacin, showing a further ∼3-log reduction in planktonic cells compared to ciprofloxacin alone, whilst an additional ∼1-log reduction was observed in biofilm-residing cells (Fig. 6A). After 2 h of treatment with 4 % w/v T-EDTA, ∼3-log reductions in viable cells in both the supernatant and the biofilm were also observed (Fig. 6B). NONOates alone showed no effect on viable cells after 2 h of treatment (Fig. 6B); however, in combination with ciprofloxacin they showed a significant 1-log increase (P ≤ 0.05) in the number of viable cells in the supernatant compared to ciprofloxacin-only treatment, but no difference in the viability of biofilm-residing cells (Fig. 6A).

## 4 Discussion

This study aimed to assess the degradation kinetics and biofilm-disrupting potential of candidate NONOates, when applied under wound-relevant temperature and pH. Additionally, the potential synergistic effects of NONOates and T-EDTA with conventional antimicrobial agents, such as ciprofloxacin, were evaluated to explore their therapeutic applicability. The findings provide insights into the pH- and temperature-dependent behaviour of NONOates and highlight critical differences in their efficacy based on biofilm maturity and metabolic activity.

### 4.1 Characterisation of pH-dependent NONOate degradation

#### 4.1.1 A higher concentration of nitrite was generated from NONOates as pH was decreased

The initial objective was to confirm NO release from SP-NO and PA-NO and evaluate how release profiles varied across wound-relevant pH environments. The Griess assay successfully detected nitrite ions in the buffer solution 30 min after the candidate NONOates were introduced (Fig. 1A), providing qualitative evidence of NO release.

Testing included pH ranges from 5.5 to 7.5 and temperatures reflective of the wound environment (35 °C), based on prior reports of chronic wound conditions [34,35]. No significant difference was observed in the total nitrite generated by SP-NO and PA-NO across pH levels (Fig. 1A). However, nitrite concentrations differed significantly between pH 5.5 and 7.5, consistent with reports of rapid, acid-catalysed NONOate decomposition at lower pH [36]. These findings underscore the pH-dependence of NONOate degradation, highlighting the importance of testing under wound-relevant temperature and pH to assess their therapeutic potential.

#### 4.1.2 Candidate NONOates showed favourable degradation kinetics at pH 7.5 and a wound-relevant temperature

The kinetic degradation profiles of NONOates support the results obtained using the Griess assay, as it was found that the NONOates at pH 5.5 had fully degraded either immediately or after 10 min, for PA-NO and SP-NO, respectively (Fig. 1B and C). PA-NO had fully degraded before the reading could be taken, whereas for SP-NO, the final ∼20 % of decomposition was observed in the first 10 min of the study. At pH 7.5, a large discrepancy was observed in the half-life values calculated from the degradation curves and those reported previously: ∼70 and 15 min (for PA-NO) and ∼277 and 39 min (for SP-NO), here and from the manufacturer, respectively [24,25]. There may be a number of reasons for this difference, with the most likely being temperature. NONOate decomposition has been shown to be highly temperature-sensitive, showing differences in the degradation rate of up to 9-fold between 22 and 37 °C [36]. Since the present study investigated decomposition rates at 32 °C, and the data provided by the manufacturer had been taken at 37 °C, this 5 °C difference in temperature may account for a large proportion of the observed discrepancy.

The elucidated NONOate degradation profiles informed experiments assessing antibiofilm efficacy. Although it was hypothesized that pH 7.5 would provide the greatest biofilm dispersal, owing to its favourable degradation and NO release profile, antibiofilm efficacy was assessed in varying pH environments with the aim of potentially decreasing the treatment timeframe.

### 4.2 NONOate-mediated biofilm dispersal

#### 4.2.1 PA-NO showed significant biofilm dispersal at pH 7.5 and a wound-relevant temperature

To assess the effects of pH and NONOate degradation on antibiofilm efficacy, 24-h *P. aeruginosa* PAO1 biofilms were treated with candidate NONOates in various pH environments at the wound-relevant temperature of 32 °C, for the first time [34,37]. PA-NO achieved the most significant reduction in biomass at pH 7.5 (Fig. 2A). The results demonstrated a significant effect of pH on NONOate-mediated antibiofilm efficacy, as no biofilm dispersal was observed after treatment with either candidate NONOate at pH 5.5 or pH 8.5. These results agree with the degradation profiles discussed previously (Fig. 1B and C), which concluded that NONOate degradation was near-instantaneous at pH 5.5, owing to their acid-catalysed decomposition mechanism. As pH was increased, the rate of decomposition decreased and NO release was more sustained, with PA-NO releasing ∼85 % of its NO at pH 7.5 but only ∼10 % at pH 8.5, within 2 h. This supports the absence of antibiofilm effects at pH 5.5, as all available NO was likely released and degraded before it could reach the bacteria owing to its short half-life. Conversely, the more sustained and slower degradation of PA-NO observed at pH 7.5 may have allowed NO to reach the target cells continually throughout the treatment period, therefore causing a more significant dispersal event. Whilst at pH 8.5, a lower concentration of NO was released from PA-NO, supporting reduced biofilm dispersal at this pH level. Another interesting observation was the lack of dispersal activity of SP-NO, as no significant dispersal was observed at any pH level at 32 °C and counterintuitively a significant increase in biofilm biomass was observed at pH 8.5. Although the exact mechanism remains unclear, this increase in biomass was not entirely unexpected, as it has been reported in the literature that NO can promote biofilm growth at certain concentrations [10]. Although a reduction in biofilm of 23 % was observed at pH 7.5, the variability between replicates was too large to provide a statistical difference. This variability in performance has remained a consistent characteristic of SP-NO throughout the present study, potentially caused by interactions between the spermine backbone of SP-NO with the biofilm or bacteria. Notably, previous literature has reported that spermidine (a comparable molecule to spermine) increases biofilm production and decreases antimicrobial tolerance by interacting with the bacterial membrane [38]. Further investigation is required to confirm any interactions between spermine and bacteria/biofilm.

Whilst topical application of SP-NO and PA-NO has not been extensively studied for dermal toxicity, one phase III clinical study applied an NO-releasing gel topically and reported low adverse event rates; the most commonly reported being mild pain and erythema at the application site [39]. Further work should consider uptake of NONOates or their metabolites (i.e. NO and the polyamine backbone) from the wound site into the systemic circulation; however, the risk of systemic toxicity is low owing to the diffusional barrier presented by biofilms and the often poorly vascularised nature of chronic wounds [40].

#### 4.2.2 NONOates showed no antibiofilm efficacy on mature biofilms, whilst T-EDTA remained effective regardless of biofilm maturity

To facilitate comparison with current literature, the treatment incubation temperature was increased to 37 °C. Whilst a previous study reported 250 μM SP-NO for 2 h to be the most effective treatment, the pH of the treatment solution was not specified [18]. Owing to the favourable degradation profiles (Fig. 1B and C) and antibiofilm effects of NONOates (Fig. 2), pH 7.5 was selected for further studies. Additionally, 4 % w/v T-EDTA was included as an alternative antibiofilm agent, as this formulation is clinically approved for use in venous lock catheters in Canada and has shown antimicrobial and antibiofilm efficacy, whilst boasting an excellent safety profile [20,21,[41], [42], [43], [44], [45]]. Topical application of EDTA at similar concentrations is reportedly well-tolerated with only mild skin irritation reported in some cases [42], whereas systemic use requires much higher concentrations, which are associated with potential adverse effects such as hypocalcaemia and nephrotoxicity [46].

To ensure selection of the most clinically-viable therapeutic candidate, antibiofilm efficacy was assessed on both 24- (i.e. early-stage) and 48-h (i.e. mature) biofilms. It was hypothesized that mature biofilms would exhibit increased tolerance to antibiofilm and antimicrobial intervention, owing to both their increased matrix density and decreased cellular activity.

Significant biofilm dispersal was observed after treatment with 4 % w/v T-EDTA irrespective of the degree of biofilm maturity, showing 56 % and 64 % biomass reductions for 24- and 48-h biofilms, respectively (Fig. 3, Fig. 4B); this agreed with the literature [20,22]. Whilst 250 μM PA-NO showed a mean reduction of 22 % against 24-h biofilms, and 250 μM SP-NO showed no efficacy (Figs. 3A and 4C and D). It was concluded that the observed efficacy was owed to NO-mediated biofilm dispersal and not the buffer solution used, as a significant difference was found between treatment groups and the pH control (Fig. S3). Interestingly, neither candidate NONOate induced significant biofilm dispersal against 48-h biofilms (Fig. 3B).

Whilst the relationship between NONOate efficacy and biofilm maturity has not been previously assessed systematically, previous work has demonstrated the increased tolerance of bacteria to antibiotics in mature biofilms. For example, Chen et al. observed that 24-h *P. aeruginosa* (PA14) biofilms were significantly more susceptible to tobramycin exposure compared to 72-h biofilms; 10 μg/mL tobramycin treatment for 1 day eradicated all bacteria in 24-h biofilms, whilst 72-h biofilms required 80 μg/mL for at least 2 days [47]. It was hypothesized that the lack of biofilm dispersal from NONOates in 48-h biofilms in the present study was a result of reduced cellular activity, as NO relies on functioning metabolism to trigger dispersal (specifically targeting intracellular phosphodiesterase enzymes), whilst the efficacy of T-EDTA was conserved regardless of biofilm maturity as EDTA's mechanism of action targets the divalent cations in the EPS matrix rather than the cells.

#### 4.2.3 The reduced efficacy of NONOates against mature biofilms was likely owed to a lower metabolic rate in P. aeruginosa cells

To further investigate this hypothesis, differences in cellular activity were evaluated by comparing the number of viable cells and the metabolic activity of cells in early-stage and mature biofilms. The results showed ∼4.5-fold lower metabolic activity and ∼3.5-fold lower CFUs in 48-h biofilms compared to 24-h biofilms (Fig. 5); this confirmed the hypothesis that the lack of NONOate efficacy was likely caused by a decrease in cellular activity. It is known that decreased cellular activity in biofilms is one primary driver of biofilm-associated antimicrobial resistance, as many of the antibiotic targets rely on unhindered metabolism to elicit their effect.

It was unclear whether the decreased efficacy was caused by the presence of a biofilm or simply by nutrient deficiency associated with a longer incubation period. To assess this, half of the growth media was replenished with fresh LB after 24 h to ensure nutrient deficiency was no longer playing a role. After the media change, the metabolic activity of 48-h biofilms showed no significant difference to that observed in 24-h biofilms (Fig. 5A), confirming that nutrient deficiency in the 48-h biofilm was the primary cause of the decreased number of viable cells and reduced metabolic rate. To determine whether the bacteria in 48-h biofilms with unchanged media were primarily dormant or dead (after 48-h incubation), the cells were revived overnight in fresh LB to allow any dormant cells to resume normal cellular processes. Revival in fresh media was successful as after 24 h, 48-h metabolism-deficient biofilms showed CFUs comparable to that observed in 24-h biofilms (Fig. 5B). This indicated that dormancy was playing a significant factor as cells returned to normal metabolic activity after they regained access to a carbon source, although a combination of dead and dormant cells was likely, as reported in a previous study [48]. An additional difference between 24- and 48-h biofilms was the ∼30 % lower crystal violet absorbance in the untreated biofilm at 48 h compared to 24 h, indicating less biofilm present in the well. This may provide another indication that nutrient deficiency was causing starvation, as a previous study has reported ∼10-fold reduction in biovolume of *P. aeruginosa* biofilms after 48 h in M9 media compared to an unstarved biofilm [48]. It was hypothesized that as biofilm-residing cells were being starved, they triggered dispersal as a stress response, hence lower CV absorbance values were observed at 48 h. This may also provide a further explanation for the lack of NONOate-mediated dispersal, as the NO signalling pathway may have already been activated and therefore exogenous NO had no added dispersal effect.

### 4.3 Combining antibiofilm treatment with antibiotic therapy

#### 4.3.1 Ciprofloxacin alone showed some efficacy against biofilm-residing bacteria

The antimicrobial effects of combination treatments against 24-h biofilms were evaluated by assessing the total number of viable cells. Viable cells in both the treatment supernatant (i.e., planktonic cells) and those residing within the biofilm were assessed to provide information on the antimicrobial efficacy against both the bacteria that had been dispersed (i.e., present in the supernatant) and those still embedded within the biofilm. It was observed that ciprofloxacin alone had significantly greater efficacy compared to the untreated control against both the planktonic and biofilm-residing cells (Fig. 6A). This observation was expected against planktonic bacteria as the MIC was expected to reduce bacterial viability by 3-log (i.e., 99.9 %); however, the efficacy against biofilm-residing bacteria was not fully anticipated as the reduced ability of ciprofloxacin to penetrate the biofilm has been reported previously [49].

#### 4.3.2 T-EDTA improved the antimicrobial effects of ciprofloxacin, whilst NONOates reduced antibiotic efficacy

After a 2-h treatment with T-EDTA, ∼3-log reductions were observed against both biofilm-residing and planktonic cells, indicating potent antimicrobial efficacy within the 2-h pre-treatment phase (Fig. 6B). Since the clinically-relevant dose used here (4 % w/v) is equal to the reported MBEC [20] and four-times greater than the MIC (Fig. S2), the observed eradication was expected as biofilm degradation and antimicrobial effects were likely occurring simultaneously. When biofilms were treated with T-EDTA for 2 h and then subsequently treated with ciprofloxacin for 24 h, further ∼3- and 1- log reductions were observed in planktonic and biofilm-residing bacteria, respectively, compared to those treated with ciprofloxacin alone (Fig. 6A). This improved efficacy of ciprofloxacin and T-EDTA in combination was likely owed to targeting bacteria using two agents with distinct mechanisms of actions: T-EDTA acting to disrupt the cell membrane and ciprofloxacin inhibiting DNA replication.

As expected, neither PA-NO nor SP-NO showed antimicrobial activity after 2 h of treatment (Fig. 6B). Whilst NO has been reported to elicit antimicrobial efficacy at higher concentrations (i.e., >1 mM), it was herein expected to be below the required concentration, even though the concentration of NO released from NONOates could not be directly measured. It was, however, expected that some potentiation of ciprofloxacin efficacy might be observed when a NONOate pre-treatment phase was included. Counterintuitively, the NONOate and ciprofloxacin combinations caused an increase in viable cells compared to ciprofloxacin monotherapy (Fig. 6A). The role of NO in bacterial metabolism could provide an explanation for this lack of potentiation. NO is produced by the host immune response (via activating inducible NO synthase (iNOS)) to bacterial infection and elicits bactericidal and bacteriostatic effects by modulating bacterial energetics, such as inhibiting cell respiration [50]. The role of NO in bacterial energetics is complex and conflicting reporting perpetuates the lack of clarity. Whilst some studies have observed antibiotic potentiation in combination with NO [16,51,52], others have reported the exact opposite observing an increased tolerance to some antibiotics after exposure to NO [[53], [54], [55]]. Bactericidal antibiotics, such as β-lactams, aminoglycosides, and quinolones have distinct mechanisms of action but all rely, in some part, on the production of reactive oxygen species (ROS) to cause cell death [56]. ROS, such as hydroxyl radicals, are potent cytotoxins and readily damage bacterial proteins, membrane components, and DNA [57]. As NO is a respiratory inhibitor, many metabolic processes responsible for production and repair of ROS targets are downregulated, essentially inhibiting antibiotic molecules from binding to one of their sites of action.

The mechanisms underpinning the conflicting observations of both NO-mediated antibiotic potentiation and attenuation remain unknown. Further work could evaluate other antibiotic classes, especially bacteriostatic agents such as tetracyclines and macrolides. It would also be interesting to assess the genetic differences in the bacteria pre- and post-exposure to NO, to further elucidate the underlying mechanisms responsible for the observed lack of efficacy.

## 5 Conclusion

Here, the suitability of two candidate NONOates (PA-NO and SP-NO) and T-EDTA (as both mono- and adjuvant therapies) was assessed for application in chronic wound infections. Using *in-vitro* biofilm assays, we evaluated the ability of NONOates to reduce biofilm biomass after application at wound-relevant pH environments (pH 5.5–8.5) and temperatures (32 and 37 °C) for a clinically applicable treatment duration (2 h). The findings suggest that while NONOates exhibit promising antibiofilm activity in early-stage biofilm formation, their efficacy diminishes significantly in well-established, mature biofilms. Additionally, whilst SP-NO lacked efficacy in all experiments, PA-NO demonstrated efficacy exclusively at pH 7.5 (at both 32 and 37 °C), among the pH levels investigated, likely suggesting a limited range of applicability. Interestingly, pre-treatment with NO appeared to induce a protective effect on the bacteria, as a reduction in ciprofloxacin efficacy was observed compared to monotherapy. This suggests a possible interaction between NO and bacterial defence mechanisms against the antibiotic used. Further work should explore the underlying mechanisms of this NO-mediated protection and consider a broader range of antibiotics to identify more suitable combinations for clinical use. T-EDTA, however, showed consistent antibiofilm efficacy regardless of biofilm maturity, and potentiated ciprofloxacin efficacy.

These results highlight the challenges in developing effective antimicrobial strategies against chronic wound infections, particularly when biofilms are present. Understanding the limitations of novel treatments, such as NONOates, is crucial for advancing therapeutic interventions and improving patient outcomes in the management of chronic wounds. T-EDTA showed therapeutic promise and future research efforts should focus on safety and the presence of side effects after topical application. Additionally, further research is needed to optimize delivery mechanisms and explore potential synergies with other antimicrobial agents to enhance NONOate-mediated disruption of biofilms in chronic wound settings. Future studies should also consider increasing the complexity of the growth media to account for the presence of proteins and enzymes, which are responsible for increased proteolytic activity that may influence biofilm formation and therapeutic efficacy.

## CRediT authorship contribution statement

**Aaron Crowther:** Writing – review & editing, Writing – original draft, Visualization, Validation, Methodology, Investigation, Formal analysis, Data curation, Conceptualization. **Gareth LuTheryn:** Writing – review & editing, Supervision, Methodology, Conceptualization. **Ramón Garcia-Maset:** Writing – review & editing, Supervision, Methodology. **Maryam Parhizkar:** Writing – review & editing, Supervision, Project administration. **J. Mark Sutton:** Writing – review & editing, Methodology. **Charlotte Hind:** Writing – review & editing, Methodology. **Dario Carugo:** Writing – review & editing, Supervision, Resources, Project administration, Methodology, Funding acquisition, Conceptualization.

## Declaration of competing interest

The authors declare the following financial interests/personal relationships which may be considered as potential competing interests: Dario Carugo reports financial support was provided by 10.13039/501100000266Engineering and Physical Sciences Research Council. If there are other authors, they declare that they have no known competing financial interests or personal relationships that could have appeared to influence the work reported in this paper.

## Data Availability

Data will be made available on request.
