# Supplementary material for: Efficacy of nitric oxide donors and EDTA against Pseudomonas aeruginosa biofilms: Implications for antimicrobial therapy in chronic wounds
Source: Biofilm. 2025 Apr 15;9:100280. doi: 10.1016/j.bioflm.2025.100280 (PMC12056783; doi:10.1016/j.bioflm.2025.100280)
Supplement: Multimedia component 1 [file mmc1.docx]

##
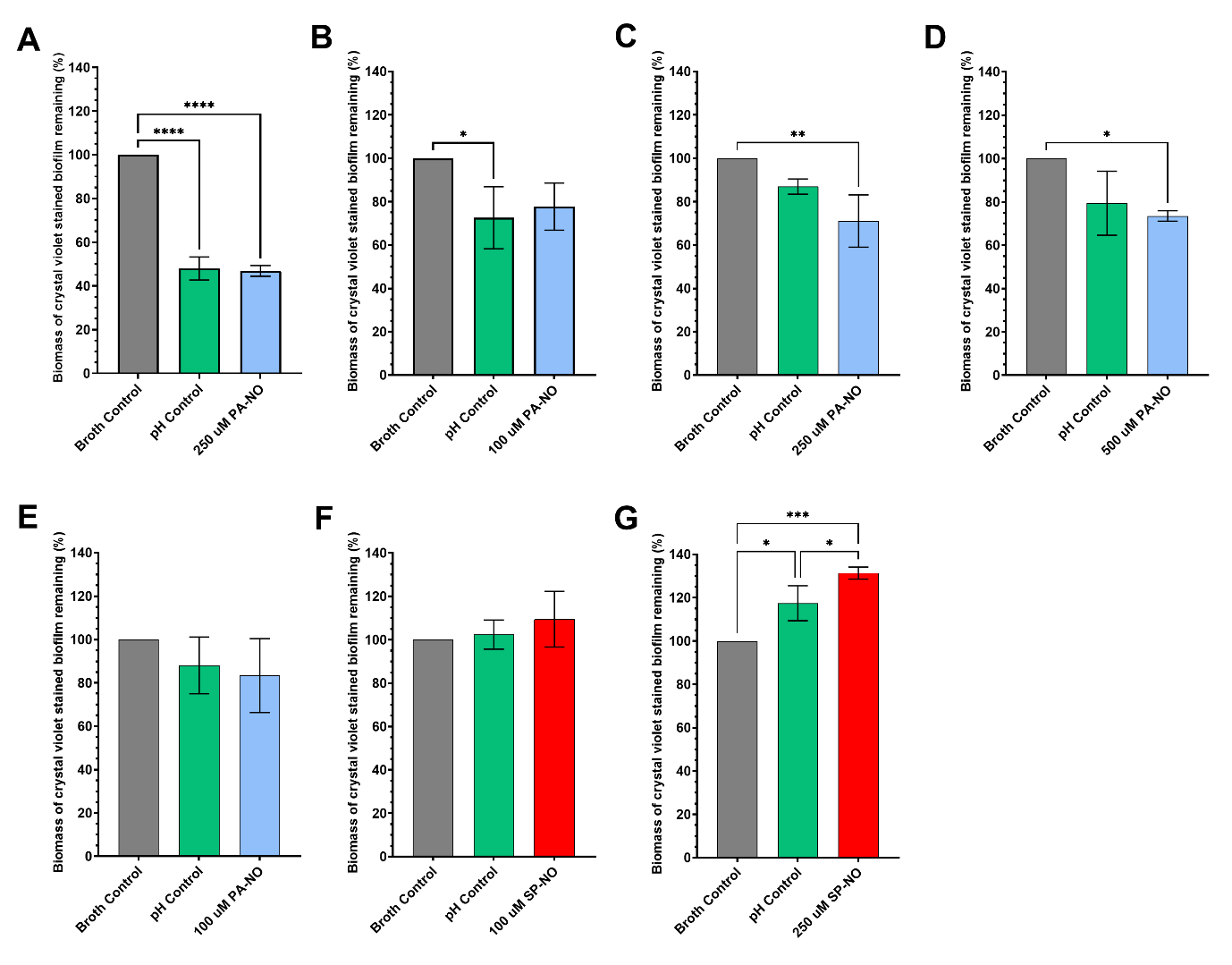
Supplementary material for “Efficacy of nitric oxide donors and EDTA against Pseudomonas aeruginosa biofilms: Implications for antimicrobial therapy in chronic wounds”

Figure S1 Assessment of antibiofilm activity of PAPA NONOate (PA-NO; blue bars) and spermine NONOate (SP-NO; red bars) against 24-hour P. aeruginosa PAO1 biofilms, for 60 min. Treatment conditions were (A) 250 µM PA-NO at pH 5.5, (B) 100 µM PA-NO at pH 7.5, (C) 250 µM PA-NO at pH 7.5, (D) 500 µM PA-NO at pH 7.5, (E) 100 µM PA-NO at pH 8.5, (F) 100 µM SP-NO at pH 7.5, and (G) 250 µM SP-NO at pH 7.5. Controls of LB broth (grey bars) and a pH control (green bars) using the same buffer solvent as the NONOates were included. Samples were incubated at 32 °C. Error bars indicate standard deviation of the mean. Experiments were carried out in triplicate (n=3).


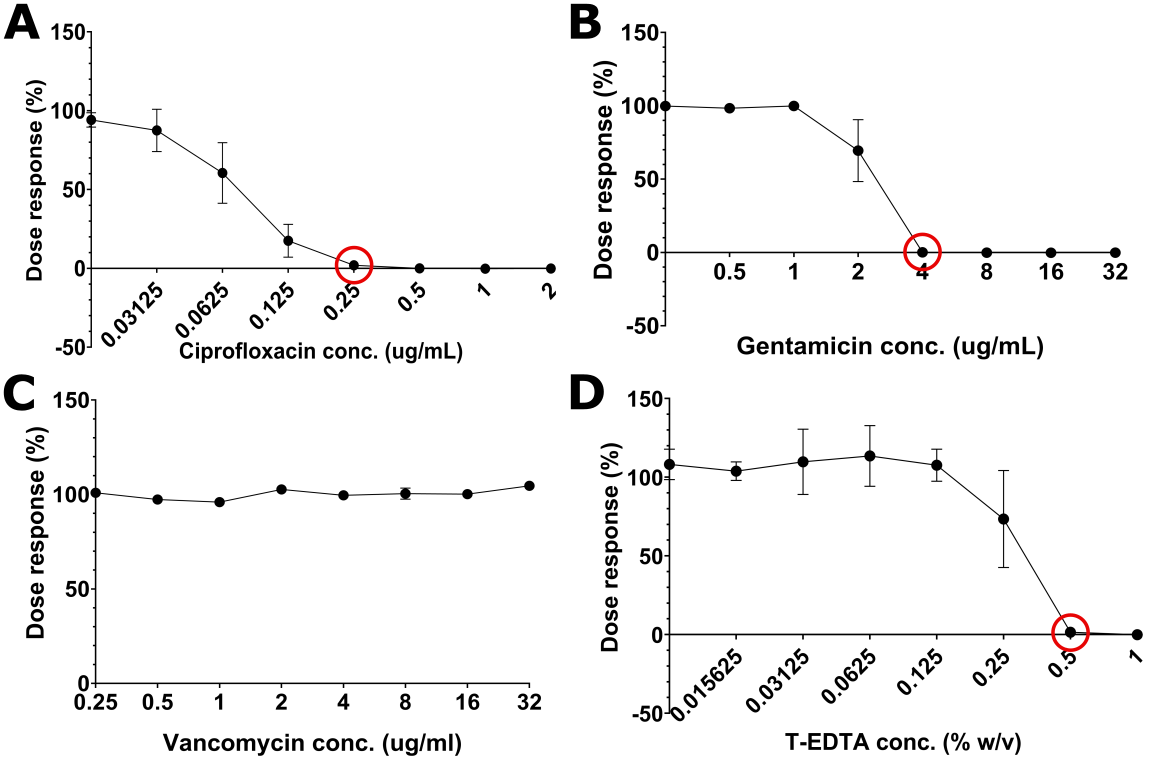


Figure S2 Minimum inhibitory concentrations (MICs) (red circles) determined by broth microdilution against Pseudomonas aeruginosa (PAO1) for (A) ciprofloxacin, (B) gentamicin, (C) vancomycin, and (D) tetrasodium EDTA (T-EDTA).


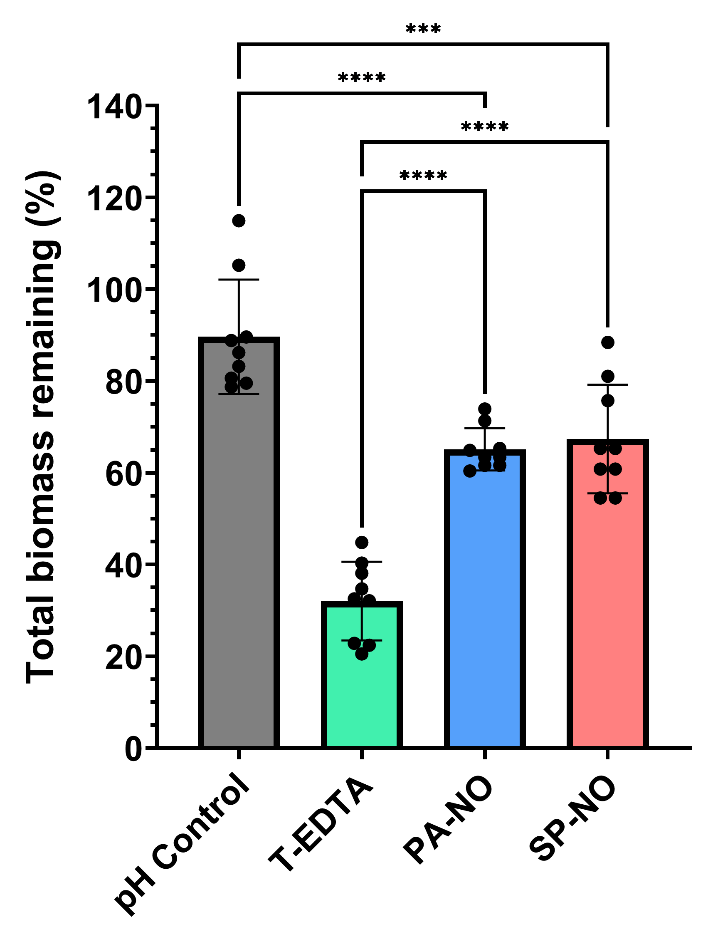


Figure S3 Percentage of total biofilm biomass remaining (crystal violet; compared to untreated) of P. aeruginosa PAO1 biofilms grown for 24 hours treated for 2 hours with either 4% w/v tetrasodium EDTA (T-EDTA; green bar), 250 µM PAPA NONOate (PA-NO; blue bar), or 250 µM Spermine NONOate (SP-NO; red bar). The pH control (grey bar) was supplemented with a 1:1 solution of LB broth plus PBS (i.e. the same buffer solution used in treatment groups) for 2 hours. Samples were incubated at 37 °C. One-way ANOVA was carried out for statistical comparison. Statistical differences are indicated by: * = p < 0.05, ** = p < 0.01, *** = p < 0.001, and **** = p < 0.0001. Error bars indicate standard deviation of the mean. Experiments were carried out with 3 biological replicates and 3 technical replicates (n=9).
